# Effect of XingPiJieYu decoction on spatial learning and memory and cAMP-PKA-CREB-BDNF pathway in rat model of depression through chronic unpredictable stress

**DOI:** 10.1186/s12906-016-1543-9

**Published:** 2017-01-24

**Authors:** Chunye Wang, Jianyou Guo, Rongjuan Guo

**Affiliations:** 1 Dongfang Hospital Beijing University of Chinese Medicine, 100078 Beijing, China; 2 CAS, Key Laboratory of Mental Health, Institute of Psychology, Chinese Academy of Sciences, 100101 Beijing, China

**Keywords:** Chinese herbs, Depression, Learning and memory, Chronic unpredictable stress, CAMP, PKA, CREB, BDNF

## Abstract

**Background:**

Depression is a mental disorder characterized by a pervasive low mood and loss of pleasure or interest in usual activities, and often results in cognitive dysfunction. The disturbance of cognitive processes associated with depression, especially the impairment of learning and memory, exacerbates illness and increases recurrence of depression. XingPiJieYu (XPJY) is one of the most widely clinical formulas of traditional Chinese medicine (TCM) and can improve the symptoms of depression, including learning and memory. However, its regulatory effects haven’t been comprehensively studied so far. Recently, some animal tests have indicated that the cyclic adenosine monophosphate (cAMP)-protein kinase A (PKA)-cAMP response element-binding protein (CREB)-brain derived neurotrophic factor (BDNF) signaling pathway in hippocampus is closely related to depression and the pathogenesis of cognitive function impairments. The present study was performed to investigate the effect and mechanism of XPJY on depression and learning and memory in animal model.

**Materials:**

The rat model of depression was established by chronic unpredictable stress (CUS) for 21 days. The rats were randomly divided into six groups: control group, CUS group, CUS + XPJY (1.4 g/kg, 0.7 g/kg and 0.35 g/kg) groups, and CUS + sertraline (10 mg/kg) group. The sucrose preference, open field exploration and Morris water maze (MWM) were tested. The expression of cAMP, CREB, PKA and BDNF protein in hippocampus was examined with Elisa and Western Blot. The mRNA level of CREB and BDNF in hippocampus was measured with PCR.

**Results:**

The results demonstrated that rats subjected to CUS exhibited decreases in sucrose preference, total ambulation, percentage of central ambulation, rearing in the open field test and spatial performance in the MWM. CUS reduced the expression of cAMP, PKA, CREB and BDNF in hippocampus of model rats. These effects could be reversed by XPJY.

**Conclusion:**

The results indicated that XPJY can improve depression and related learning and memory and the effect of XPJY is partly exerted through the cAMP-PKA-CREB-BDNF signaling pathway.

## Background

Depression is a mental disorder characterized by a pervasive low mood and loss of pleasure or interest in usual activities [1] and is the most common psychiatric illness that involves the disturbance of mood, with 10 to 20% lifetime prevalence [2]. The patients also demonstrate sleeplessness and suicidal tendencies, decreased food-intake and body-weight [3, 4], and more importantly always suffer from obvious cognitive function impairments, such as delayed thinking correlation, reduced computational capability, learning and memory impairment, and reduction in attention, comprehension and judgment [5–7]. Learning and memory impairment is one of the important residual symptoms, which has a strong impact on function of patients both at home and workplace. On the other hand, it is becoming increasingly clear that the disturbance of cognitive processes, especially the impairment of learning and memory, plays an important role in the development and complete recurrence of depression [8, 9]. First of all, late-life depression is a risk factor for cognitive decline [10]. Furthermore, cognitive impairment is one of the typical features of recurrent depressive disorder (rDD), predominantly connected with episodic memory processes and the frontal functions (working memory) [11–13]. Cognitive impairment, linked with the earlier onset of depressive symptoms and episode prolongation, may in return lead to an ineffective antidepressant therapy and impede full recovery [14]. So it is the demand of reality and also an important task not to be avoided by mechanism research.

At present, depression is commonly treated with monoamine-based antidepressants such as selective serotonin reuptake inhibitors (SSRI). However, about 1/3 of depressive patients are not responsive to conventional antidepressants [15], and these drugs is associated with a delay in symptom remission [16], severe side effects [17] and unsatisfactory outcome in cognition. Therefore, the treatment of depression is still a big problem and requires more effective approaches.

Traditional Chinese Medicine (TCM) has a long history in preventing and treating depression in Asia and is receiving more and more attention [18–22]. XPJY is a common prescription in clinical practices and shows relatively satisfactory therapeutic effect in improving depression and learning and memory level. Previous studies have shown that XPJY could improve behaviour and antidepression in chronic unpredictable stress model of depression in rats, which was as good as sertraline, and it also could reverse serum 5-HT and corticosterone from depression rats. Another study found that XPJY has the better effect on improving learning and memory ability in depression rats than sertraline, which might he related to reduce the inflammatory factors level, such as IL-1β, IL-6 and TNF-α, in serum and hippocampus. Recently, some animal tests have indicated that the cyclic adenosine monophosphate (cAMP)-protein kinase A (PKA)-cAMP response element-binding protein (CREB)-brain derived neurotrophic factor (BDNF) signaling pathway in hippocampus is closely related to depression and the pathogenesis of cognitive function impairments [23–26]. However, the molecular mechanisms remain unclear and require verification in animals. A further study should be done this time. XPJY was composed of the following dried raw materials: acori tatarinowii rhizoma (Shi-Chang-pu), American ginseng (Xi-Yang-shen), radix curcumae (Yu-Jin) and prepared rehmannia root (Shoo-Di-huang). All of these were produced in accordance with the China Pharmacopoeia standard of quality control.

In the present study, we investigated the effects of XPJY on rats with depression established by chronic unpredictable stress and on the expression of cAMP-PKA-CREB-BDNF signal pathway.

## Methods

### Preparation and compositional analysis of XPJY

**Fig 1:**
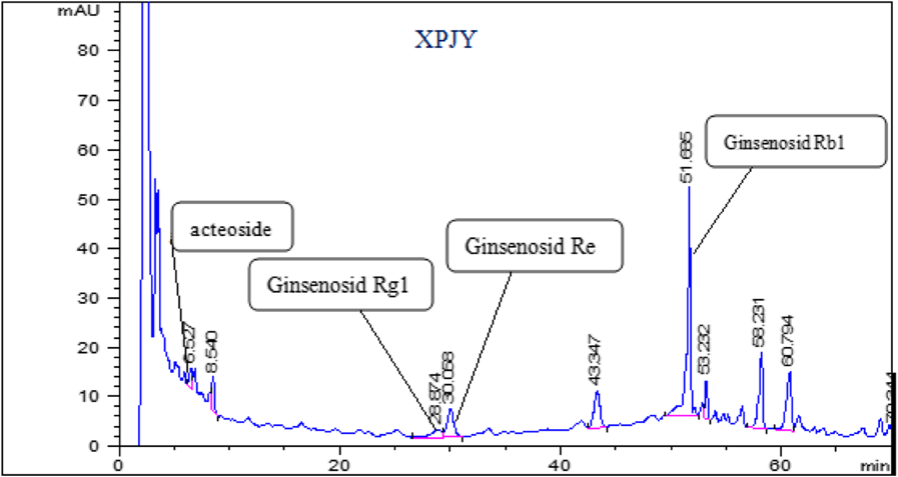
The HPLC Chromatogram of XPJY

XPJY was composed of the following granule, which were derived from dried raw materials, including acori tatarinowii rhizoma (Shi-Chang-pu), American ginseng (Xi-Yang-shen), radix curcumae (Yu-Jin) and prepared rehmannia root (Shoo-Di-huang), in weight ratio of 0.5:1.2:2:3. All granules were bought from medicinal Materials Company of Beijing KangRenTang Company (Beijing, China), and analyzed for composition by high performance liquid chromatography (HPLC). The test solution was prepared by dissolving XPJY in methanol, and analyzed on an Agilent 1200 HPLC system with an C18 analytical column (250 × 4.6 mm, 5 μm). The mobile phase was consisted of water and acetonitrile in gradient elution. For quality control, the ginsenosides Rg1, Re, Rb1 and acteoside were used as standard constituents. All the standards were purchased from National Institutes for Food and Drug Control (Beijing, China). As shown in Fig. 1, four bioactive compounds including ginsenosides Rg1, Re, Rb1 and acteoside were found and exhibited high stability in XPJY by HPLC.

### Animals and grouping

Adult male Sprague–Dawley rats (200–220 g) provided by Beijing Academy of Military Medical Sciences Laboratory Animal Centre were individually kept on a 12:12 h light/dark cycle in home cages with food and water available ad libitum except as described in stress. The experiment was approved by the Animal Ethic Committee of the university. Sixty rats (10 rats in each group) were randomly divided into six groups: control group receiving once-daily oral gavage (PO.) administration of distilled water for 21 days, CUS group receiving CUS and once-daily PO. administration of distilled water for 21 days, CUS + sertraline group receiving CUS and once-daily Po. sertraline at 10 mg/kg (Pfizer Inc., USA) for 21 days, CUS and XPJY groups receiving CUS and once-daily PO. XPJY at 0.35, 0.7 and 1.4 g/kg, respectively, for 21 days. The sertraline diluted in distilled water and XPJY were orally given 1 h before CUS exposure. The dosage “0.7 g/kg” has been calculated based mainly on the dosages of humans and animals. Then the double dosage was considered as one group, half dosage as another, hereby it works out that dose of XPJY is made as three groups: 0.35, 0.7 and 1.4 g/kg.

### Animal model preparation

The CUS procedure was modified from the procedures used by Heine et al. [27]. Briefly, rats were exposed to different stressors daily for 21 days as follows: day1, cold immobilization for 1 h at 4 °C, forced swim for 30 min at 25 °C; day 2, immobilization for 1 h, crowding for 23 h; day 3, forced cold swim stress for 5 min at 10 °C, isolation for 23 h; day 4, immobilization for 1 h, vibration for 1 h; day 5, forced swim stress for 30 min at 25 °C, cold immobilization for 1 h at 4 °C; day 6, forced cold swim stress for 5 min at 10 °C, crowding for 23 h; day 7, vibration for 1 h, isolation for 23 h. This schedule was repeated twice for a total of 21 days. Prior to the study, certain criteria were set for excluding animal on weight loss, or the possible occurrence of wounds. Rats were acclimated to 3 min of handling once a day for 7 consecutive days before being used in experiment and were weighed on the 1st and 21th day of handling.

### Sucrose preference test

Sucrose preference test was used to define anhedonia as a reduction in sucrose intake and sucrose preference. The sucrose preference test consisted of firstly removal of the food and water from the cage for a period of 20 h. Then water and 1% sucrose were placed back to the cage and animals were allowed to consume the fluids freely for a period of 1 h. The preference test was performed twice, separated by at least 5 days to calculate the mean for baseline. Then the preference test was conducted following the 21 days CUS period. On the last stress day, rats were deprived of water and food for 20 h and from the next day were given 1 h sucrose preference test (24 h after the last drug treatment). Sucrose and water consumption (ml) was measured and the sucrose preference was calculated as the sucrose preference (%) = sucrose consumption/(sucrose consumption + water consumption).

### Open field exploratory behavior test

Open field test was used to study the exploratory and anxiety behaviors of rats and was performed after the sucrose preference test. The rat was placed in the central square and observed for 5 min by a video camera and taped for further analysis. Motility was scored when the rat crossed a sector border with both hind-limbs. The following behaviors were scored by an observer who was blind to the drug treatment: central ambulation, number of central squares crossed; total ambulation, the overall number of peripheral and central square crossed; rearing, number of times the animal stood on its hind limbs; grooming, number of times the animal made these responses viz. grooming of the face, licking/cleaning and scratching the various parts of the body; immobility period, the time spent immobile. Anxiety-related behavior was measured by the percentage of central ambulation and calculated as the percentage of central ambulation (%) = central ambulation/total ambulation. Between tests, the apparatus was cleaned with 5% alcohol.

### Morris water maze test

After the final stress stimulation, the Morris water maze (MWM) was used to determine the spatial learning and memory of the rats. The behavior of the animal was monitored with a video camera mounted in the ceiling above the centre of the pool. For oriented navigation test, the rats were trained for 120 s per trial and 4 trials per day starting at four different positions with 30 min intervals for 4 consecutive days as acquisition trials. Each trial began with the rat in the pool facing the sidewalls. When the rat escaped onto the platform, the rat was allowed to stay on the platform for 30 s before being returned to home cage. If the rat failed to escape within 120 s, it was guided to the platform by the experimenter and allowed to stay for 15 s. Each rat was trained once in the morning and once in the afternoon, and the time it took to find the platform (the latency period) was recorded. If the rats could not find the platform within 120 s, they were taken out the water and the time was recorded as 120 s. For the spatial exploration test, the hidden platform was removed on day 5, and memory retrieval was examined by a probe trial that lasted for 120 s. The escape latency in the acquisition trials, the times of crossing the platform position and the time spent in the target quadrant in the probe test were recorded by a computerised video tracking system.

### Measurement of the expression level of cAMP level

After the last behavior test, hippocampus was taken out and the cAMP content was evaluated using EliteTM cAMP ELISA Assay Kit (eEnzyme, Montgomery Village, MD, USA). Extracted supernatants in each sample were loaded into EliteTM cAMP ELISA Assay Kit plates and each procedure was performed according to the manufacturer’s instructions. After color development, the absorbance was measured at Ex/Em = 540/590 nm with fluorescence reader (Bio-Rad, CA, USA).

### Measurement of PKA, CREB, BDNF protein expressions by western bolt

After the last behavior test, rats were sacrificed. Then the hippocampus was dissected, put into chilled tubes treated with an enzyme inhibitor and homogenized for Western blot analysis as previously reported [27], using primary antibodies for PKA, CREB and BDNF (1:2000, Santa Cruz Biotechnology) and β-actin (1:10000, Santa Cruz Biotechnology) followed by secondary antibodies conjugated with horseradish peroxidase (HRP, 1:5000, Bio-Rad). Immunoblots were visualized on X-ray film by chemiluminescence reaction (Pierce), and image analysis was performed on optical density calibrated images by AlphaEase Stand Alone software (Alpha Innotech). All experiments were performed 3 times.

### Evaluation of mRNA expression levels of CREB and BDNF by polymerase chain reaction (PCR)

Total RNA from the hippocampus was isolated with RNASure® Mini Kit according to the manufacturer’s protocol. The concentration of extracted RNA was calculated from the absorbance at 260 nm and the quality of RNA was assessed by absorbance at 260 and 280 nm, with an acceptable ratio of A260 to A280 ranging from 1.9 to 2.1. Total RNA (1.5 mg) was transcribed using a high capacity cDNA reverse transcription kit (The Maxima® First Strand cDNA Synthesis Kit) according to the manufacturer’s protocol. Real-time quantitative polymerase chain reaction (qPCR) analysis was performed with a Maxima® SYBR Green qPCR Master Mix (2X), using the StepOnePlusTM real-time PCR system (Applied Bio systems, Inc., Foster City, CA). The details of all oligonucleotide primer sequences are listed in Table 1. The PCR reaction system was denaturation at 95 °C for 15 min followed by 40 cycles of 94 °C for 15 s, 55 °C for 30 s and 72 °C 45 s, with final extension at 72 °C for 10 min. Sequence Detection System Software (Version 1.0, Applied Bio systems, Inc., Foster City, CA) was used for data analysis. The relative expression of BDNF and CREB mRNAs was normalized to the amount of GAPDH.

**Table 1:** Sequences of PCR primers

Gene	Primes	Nucleotide sequences5′-3′	Product size (bp)	Accession No.
BDNF	Forward Reverse	5’-TGTGCGACAGCATTAGTGAG-3’ 5’-GCGTAGTTCGGCATTGGGAG-3’	216	XM_004418547
CREB	Forward Reverse	5’-TGAGTTGGCAAGTCCATTCG -3’ 5’- AACGGGCTATCCTGGTGAGT -3’	156	NM_012922
GAPDH	Forward Reverse	5’- CGGCAAGTTCAACGGCACAG -3’ 5’- CGCCAGTAGACTCCACGACAT -3’	143	NM_017008

### Statistical analysis

The data were expressed as mean ± S.E.M and analyzed by one-way ANOVA followed by Dunnet’s multiple comparisons using GraphPad Prism 4.0. *p <* 0.05 was set as significant level.

## Results

### Body weight measurement

At the 1st day of the CUS period, rats from different groups showed no significant difference in body weight (*p* > 0.05). However, significant difference was observed among groups following 21 days of CUS as shown in Table 2 (*p <* 0.01). The CUS significantly attenuated the gain of body weight when compared to control group (*p <* 0.01). Treatment with XPJY at 0.7 and 1.4 g/kg significantly prevented the inhibition of body weight gain induced by CUS (*p <* 0.01); however, 0.35 g/kg XPJY had no effect on the body weight gain induced by CUS (*p* > 0.05). In addition, 10 mg/kg sertraline also prevented the effect of CUS on body weight gain at day 21 of treatment (*p <* 0.01; Table 2).

**Table 2:** The changes of animal body weight and sucrose preference in different groups

Groups	Body weight (g)		Sucrose preference (%)	
	1st day	21st day	1st day	21st day
Control CUS CUS + sertraline 10 mg/kg CUS + XPJY 1.4 g/kg CUS + XPJY 0.7 g/kg CUS + XPJY 0.35 g/kg	184.3 ± 5.8 183.4 ± 9.0 183.5 ± 7.8 180.6 ± 10.0 180.8 ± 13.7 181.9 ± 13.2	317.0 ± 14.8 244.0 ± 38.8++ 301.5 ± 19.2** 308.1 ± 22.0** 294.5 ± 16.6** 273.6 ± 30.1	0.865 ± 0.082 0.867 ± 0.058 0.857 ± 0.064 0.888 ± 0.108 0.878 ± 0.102 0.864 ± 0.076	0.874 ± 0.091 0.598 ± 0.117++ 0.761 ± 0.242* 0.776 ± 0.141* 0.712 ± 0.153 0.682 ± 0.186

Note: ++*p* < 0.01 vs Control group; **p < 0.01 vs CUS group; *p < 0.05 vs CUS group

### Sucrose preference tests

Before the CUS period, rats from different groups showed no significant difference in sucrose preference at day 1. The sucrose preference was significantly reduced in the CUS group relative to the control group after exposure to CUS for 21 days (*p <* 0.01). Chronic treatment with XPJY at 1.4 g/kg and sertraline at 10 mg/kg significantly suppressed the CUS-induced decrease in sucrose preference (*p <* 0.05). However, XPJY at 0.7 g/kg and 0.35 g/kg had no effect on the effect in sucrose preference induced by CUS (*p* > 0.05; Table 2).

### Open field exploratory behavior test

The result indicated CUS rats exhibited decreased total ambulation, percentage of center ambulation and rearing in comparison to control rats (*p <* 0.01 or *p <* 0.05). XPJY 1.4 g/kg and sertraline 10 mg/kg significantly reversed the CUS-induced behavioral alterations, as observed by increased total ambulation, percentage of central ambulation and rearing as compared to the CUS group (*p <* 0.05). XPJY 0.7 g/kg and 0.35 g/kg treatment didn’t affect the stress induced behavior alterations in open field test (Table 3).

**Table 3:** The effects of XPJY on open field test in different groups

Groups	Total ambulation	Central ambulation (%)	Rearing
Control CUS CUS + sertraline 10 mg/kg CUS + XPJY 1.4 g/kg CUS + XPJY 0.7 g/kg CUS + XPJY 0.35 g/kg	4749.1 ± 835.3 3834.2 ± 588.1+ 4616.4 ± 499.6* 4506.0 ± 638.5* 4335.8 ± 773.1 4144.5 ± 649.1	22.6 ± 12.7 6.3 ± 4.3++ 14.1 ± 8.7* 16.2 ± 8.1* 12.1 ± 6.8 9.7 ± 12.8	9.1 ± 3.2 3.4 ± 2.1++ 7.8 ± 3.5* 7.0 ± 3.8* 5.4 ± 3.8 5.1 ± 4.5

Note: ++*p* < 0.01 vs Control group; +*p* < 0.05 vs Control group; *p < 0.05 vs CUS group

### Oriented navigation and spatial exploration tests

The MWM test indicated that the escape latency in the CUS group was significantly prolonged compared to the control group on day 1 and 2 (*p <* 0.01). In comparison to the CUS group, treatment with XPJY 1.4 g/kg significantly shortened the escape latency on day 1 (*p <* 0.05), while the treatments with XPJY at 0.35 and 0.7 g/kg and sertraline at 10 mg/kg did not show significant effect on the escape latency induced by CUS (*p* > 0.05; Table 4). In spatial test, CUS impaired memory retrieval as indicated by fewer crossing times of the platform position, while sertraline 10 mg/kg and XPJY 1.4 g/Kg treatments restored the CUS-induced impairment of memory retrieval to levels seen in controls. XPJY 0.35 and 0.7 mg/kg had no effect on the memory retrieval impairment by CUS (*p* > 0.05; Table 4).

**Table 4:** The effects of XPJY and sertraline on escapes latency in different groups

Groups	Escape latency (s)				Times crossing platform position
	Day1	Day2	Day3	Day4	Day5
Control CUS CUS + sertraline 10 mg/kg CUS + XPJY 1.4 g/kg CUS + XPJY 0.7 g/kg CUS + XPJY 0.35 g/kg	25.2 ± 8.2 38.8 ± 7.6++ 30.5 ± 13.4 28.6 ± 9.3* 33.1 ± 6.8 34.4 ± 14.6	23.3 ± 6.7 32.9 ± 9.2+ 27.7 ± 7.3 25.3 ± 9.6 27.7 ± 10.7 28.7 ± 9.8	21.5 ± 5.9 23.5 ± 5.1 20.2 ± 4.4 20.9 ± 5.2 20.0 ± 4.2 19.6 ± 4.5	18.1 ± 4.1 18.9 ± 5.4 17.6 ± 4.3 17.5 ± 7.3 17.8 ± 5.4 18.9 ± 5.0	4.38 ± 1.51 2.13 ± 1.13++ 3.63 ± 1.41* 3.88 ± 1.64* 3.13 ± 1.13 2.88 ± 1.46

Note: ++*p* < 0.01 vs Control group, +*p* < 0.05 vs Control group; *p < 0.05 vs CUS group

### Effects of XPJY on the expression of cAMP

**Fig. 2:**
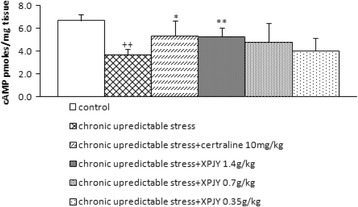
Effect of XPJY on cAMP following 21 days of chronic unpredictable stress. cAMP was reduced in the chronic unpredictable stress group compared with control group (*P* < 0.01). Chronic treatment with XPJY 1.4 g/kg significantly increase in cAMP compared with the CUS group (*P* < 0.01). Sertraline 10 mg/kg group also significantly increase in cAMP compared with the CUS group (*P* < 0.05). XPJY 0.7 g/kg and 0.7 g/kg treatment did not alter stress-induced cAMP alterations. ++*P* < 0.01, as compared to the control group; *P < 0.05, as compared to the chronic unpredictable stress group

In comparison to the control group, the expression of protein cAMP significantly decreased in the hippocampus of the rats in CUS group (*p <* 0.01). The treatments with XPJY 1.4 g/kg and sertraline 10 mg/kg significantly increased the expression of protein cAMP when compared to CUS group (*p <* 0.01 or *p <* 0.05). However, XPJY at 0.35 and 0.7 g/kg had no significant influence on the decrease of cAMP induced by CUS (*p* > 0.05; Fig. 2).

### Effects of XPJY on the expression of PKA, CREB and BDNF proteins

**Fig. 3:**
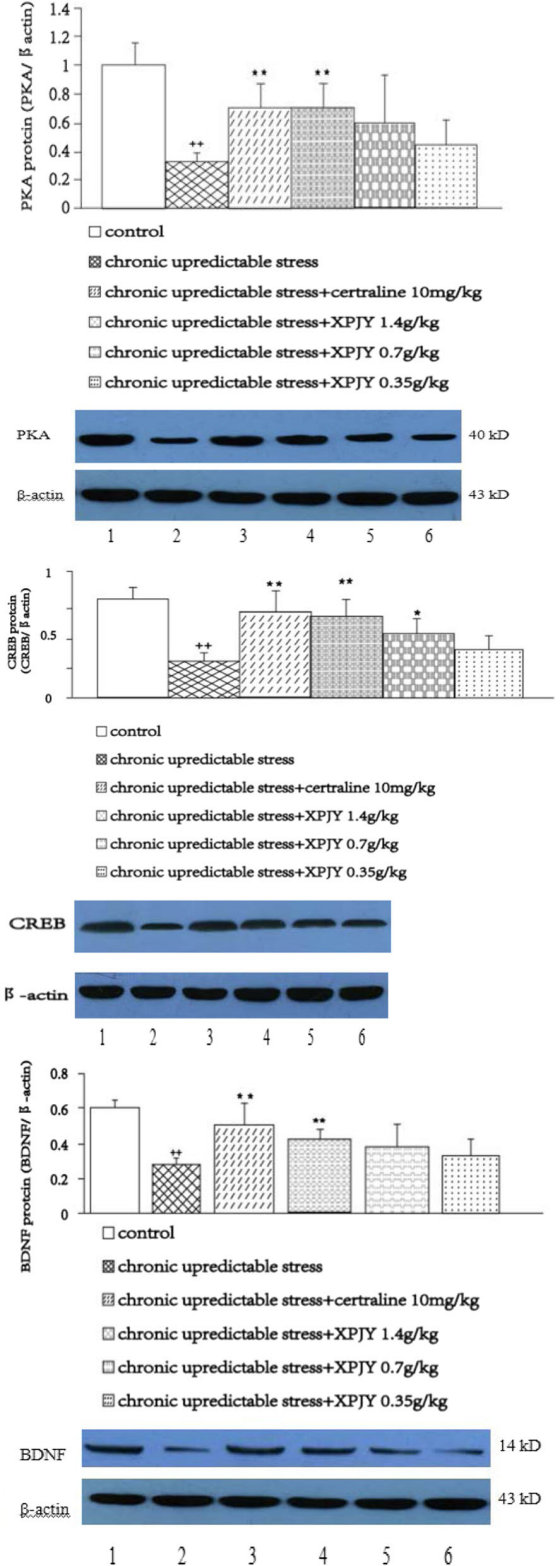
Effect of XPJY on hippocampal PKA, CREB and BDNF protein level following 21 days of chronic unpredictable stress. Relative optical density (OD) of PKA/CREB/BDNF to β-actin. PKA, CREB and BDNF were reduced in the chronic unpredictable stress group compared with control group (*P <* 0.01). Chronic treatment with XPJY 1.4 g/kg significantly increase in PKA, CREB and BDNF compared with the CUS group (*P <* 0.01). Sertraline 10 mg/kg group also significantly increase in PKA, CREB and BDNF compared with the CUS group (*P <* 0.01). XPJY 0.7 g/kg group also significantly increase in PKA, CREB and BDNF compared with the CUS group (*P <* 0.05). XPJY 0.7 g/kg and 0.35 g/kg treatment did not alter stress-induced PKA, BDNF alterations (*P* > 0.05). ++*P <* 0.01, as compared to the control group; *P < 0.05, as compared to the chronic unpredictable stress group. Representative immunoblots of PKA/CREB/BDNF and β-actin. 1, control; 2, chronic unpredictable stress; 3, chronic unpredictable stress plus chronic sertraline (10 mg/kg); 4, chronic unpredictable stress plus chronic XPJY (1.4 g/kg); 5, chronic unpredictable stress plus chronic XPJY (0.7 g/kg); 6, chronic unpredictable stress plus chronic XPJY (0.35 g/kg)

In comparison to the control group, the expression of protein PKA, CREB and BDNF significantly decreased in the hippocampus of the rats in CUS group (*p <* 0.01). The treatments with XPJY 1.4 g/kg and sertraline 10 mg/kg significantly increased the expression of protein PKA, CREB and BDNF when compared to CUS group (*p <* 0.01). The treatments with XPJY 0.7 g/kg significantly increased the expression of protein CREB when compared to CUS group (*p <* 0.05) However, XPJY at 0.35 and 0.7 g/kg had no significant influence on the decrease of PKA, BDNF induced by CUS (*p* > 0.05). XPJY at 0.35 had no significant influence on the decrease of CREB induced by CUS (*p* > 0.05; Fig. 3).

### Expression of CREB and BDNF mRNAs

**Fig. 4:**
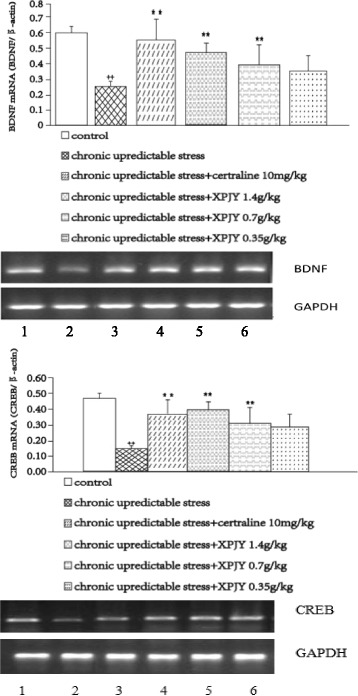
CREB and BDNF mRNA expression following 21 days of chronic unpredictable stress. 1, control; 2, chronic unpredictable stress; 3, chronic unpredictable stress plus chronic XPJY (0.35 g/kg);4, chronic unpredictable stress plus chronic XPJY (0.7 g/kg); 5, chronic unpredictable stress plus chronic XPJY (1.4 g/kg); 6, chronic unpredictable stress plus chronic sertraline (10 mg/kg). CREB and BDNF mRNA were reduced in the chronic unpredictable stress group compared with control group (*P* < 0.01). Chronic treatment with XPJY 1.4 g/kg significantly increase in CREB and BDNF mRNA compared with the CUS group (*P* < 0.01). Sertraline 10 mg/kg group also significantly increase in cAMP compared with the CUS group (*P* < 0.01). Chronic treatment with XPJY 0.7 g/kg significantly increase in CREB and BDNF mRNA compared with the CUS group (*P* < 0.01 or *P* < 0.05). XPJY 0.35 g/kg treatment did not alter stress-induced CREB and BDNF mRNA alterations. ++*P* < 0.01, as compared to the control group; *P < 0.05, **P < 0.01, as compared to the chronic unpredictable stress group

The result indicated that the expression of CREB and BDNF mRNAs was significantly decreased by CUS when compared to control group (*p <* 0.01 or *p <* 0.05). Chronic treatments with XPJY prevented the reduction of CREB and BDNF mRNA expression by CUS in a dose-dependent manner. Treatment with XPJY 1.4 g/kg and 0.7 g/kg significantly prevented the CUS-induced reduction of CREB and BDNF mRNAs expression (*p <* 0.01 or *p <* 0.05), however XPJY 0.35 g/kg had no effect (*p* > 0.05). Similarly, sertraline 10 mg/kg treatment also markedly increased the expression of BDNF mRNAs (*p <* 0.01; Fig. 4).

## Discussion

The present study utilized broadly accepted traditional animal models – CUS model to examine depressive behaviors, the spatial learning and memory capability and the expressions of mRNAs/proteins of the cAMP-PKA-CREB-BDNF signaling. The results indicated that the body weight gain, sucrose preference, total ambulation, percentage of central ambulation and rearing after CUS for 21 were significantly decreased. It indicates that the depression model was successfully established. Then the spatial cognitive performance in the Morris water maze task was also decreased, which demonstrated that depression by CUS had a dramatic influence on spatial cognitive performance in the MWN test, while the treatment of the rats with XPJY significantly reversed these changes. Furthermore, the expressions of mRNAs/proteins in the cascade of cAMP-PKA-CREB-BDNF signaling were decreased by CUS, too. The administration of XPJY to stressed rats prevented such metabolite reductions. These results suggest that XPJY could improve depression and related learning/memory impairment through the cAMP-PKA-CREB-BDNF signal cascade. Depression is a highly debilitating and widely distributed mental illness in the general population with a lifetime incidence of 15-25%, ranking it as one of the most burdensome diseases of society [5–7], besides enormous personal suffering and increased mortality risk [28]. Depression may display many different syndromes like a pervasive low mood and loss of pleasure or interest in usual activities [1]. The patients also demonstrate sleeplessness, decreased body-weight [3, 4], and obvious cognitive function impairments, such as learning and memory impairment, etc. [5–7]. Cognitive function related to depression has received far more attention. A growing body of research suggests that depressive symptoms and cognitive impairment are common often coexist in an individual patient, especially in older adults. And late-life depression even is a risk factor for cognitive decline [10]. Another clinical research also found that depressed participants, in the normal aging older adults, had a lower performance compared to non-depressed participants in cognitive domains, and the depressive symptoms may have a distinct impact on cognitive performance [29]. The studies show that the pattern of cognitive impairment associated with depressive symptoms involves executive dysfunction, reduced processing speed, and deficits in episodic memory [30–32], while global intellectual ability, language skills, visuospatial abilities, and semantic processing are usually spared [33].

Among them, learning and memory impairment is one of the important cognitive impairments and residual symptoms, which has a strong impact on function of patients both at home and workplace, and the life quality of patients. Many studies also have reported learning and memory deficits in depressed subjects [3]. Further, it is becoming increasingly clear that the disturbance of cognitive processes, especially the impairment of learning and memory, plays an important role in the development and complete recurrence of depression [8, 9]. In addition, cognitive impairment is also one of the typical features of recurrent depressive disorder, predominantly connected with episodic memory processes and working memory [11–13]. And cognitive impairment, linked with the earlier onset of depressive symptoms and episode prolongation, may in return lead to an ineffective antidepressant therapy and impede full recovery [14].

In this study, the chronic unpredictable stress for 21 days was used to establish depression model of rat and simulate the long-term negative modes and life events of humans. The chronic unpredictable stress procedure is one of the well-validated animal models of depression [34], that has good face validity in rodents as it can elicit depression like symptoms such as lack of sucrose preference [35, 36] interpreted as anhedonia [37] and reduced locomotor activity [38]. Anhedonia-like behavior is the core symptom of human depression [39], which means inability to experience pleasure. Anhedonia has been defined as decreased responsiveness to rewards [40, 41], and it is measured originally by declining intake of a palatable sweet solution. In this experimental conditions, there has been a significantly reduction of sucrose preference in CUS group compared with the control, which was reduced to approximately 27% at day 21 after the beginning of stress exposure. Reduced locomotor activity of rats in open field test may mimic some aspects of human psychomotor retardation [42], which is an accompanying symptom of depression in humans [40]. In the experimental conditions, chronic unpredictable stress rats also exhibited depressive behavior which is displayed by decreased total ambulation, central ambulation, rearing in comparison to control rats. Depression may also display many other different syndromes like decreased body weight [43] and impaired learning/memory [44].

The Morris water maze has been widely used all over the world to detecting spatial learning memory capability [45], to objectively reflect their cognitive levels. The rats are trained to learn to use the relationship between environmental labels and latent platforms in order to judge the positions of the platforms in the water. They may thus form stable spatial cognition. The rats were allowed to swim from the original position to the latent platform under water by utilizing the indications at the distal end. Their spatial learning was evaluated by repeated training. When the platforms under water were withdrawn, the reference memory of the animals was determined by using the frequency of penetration of the platform position [7]. The present study found that the escape latency in the XPJY 1.4 g/kg group on the first day was significantly shortened, but the difference for the sertraline group was not statistically significant. This results shows XPJY could increase the ability of spatial learning of depression rats by CUS. In contrast, the frequency for penetrating the central areas increased both in the XPJY 1.4 g/kg group and in the sertraline group in the spatial exploration test on the fifth day. It indicated that they can both achieve improvement with regard to rats’ memory capability, while the efficacy of the XPJY 1.4 g/kg group was more significant. This results shows XPJY increased the ability of spatial learning memory better than sertraline.

Previous studies have shown that chronic stressful life events are major reactions for inducing depression and a decrease in learning memory capability [44], as the major target for stress. The experimental results are consistent with actual situation. The primary findings of the present study show that CUS causes cognitive decline and depression like symptoms whereas XPJY showed ameliorating potential against detrimental effect of CUS. These results are in agreement with several other studies which also showed that UCMS causes cognitive decline and depression like behavior in animal models [38, 46].

The neurobiological mechanisms connecting the depressive symptoms with cognitive and functional performance are heterogeneous and have not been completely elucidated. A number of studies have shown that abnormalities in the hippocampus are closely associated with the occurrence and development of depression [47–50]. Depression may affect learning memory capability by injuring hippocampus neurons [51], which has strong connections with depression and learning memory capability [52]. The mechanisms for cognitive disorder in depression mainly have two dimensions: neurobiological and vascular factors, which may mediate the cognitive and functional changes associated with depression [53], including changes in monoamine systems dysfunction, hormonal and immunologic changes, inflammatory processes, and alterations on genes expression [54]. Such as, the hypothalamic-pituitary-adrenal axis dysfunction in depression, which relates to hippocampal atrophy, may be a neurobiological causal factor to the episodic memory impairment in depressed subjects [54, 55]; and white matter lesions as vascular burden have be found in depressed subjects [56]. Our earlier studies have shown that learning and memory ability in depression rats might be related to reduce the inflammatory factors level, such as IL-1β, IL-6 and TNF-α, in serum and hippocampus [57].

Pathophysiological studies on depression have recently been gradually transferred to the intracellular secondary messenger system. As an intracellular secondary messenger, cAMP can promote neuronal differentiation and survival [58, 59] as well as outgrowth, regeneration [60–62] and guidance of neuronal processes [63, 64], whose signaling has been shown to be implicated in mechanism of reduced synaptic plasticity, that may contribute to the pathophysiology of depression [65, 66]. cAMP can activate cAMP-dependent protein kinase (PKA), and subsequently PKA is able to activate CREB directly by phosphorylation of the transcription factor CREB [67] or indirectly [58, 68], thus further mediating multiple signaling molecules, like CREB and BDNF which play important roles in the signaling pathway of learning memory and depression [10].

CREB is a kind of regulatory factor in nuclei, as an important component of multiple intracellular signaling pathways in the nervous system, and is capable of regulating transcription by autophosphorylation. Many intracellular signal transduction cascades can influence, directly or indirectly, the activation of CREB. Some of the enzymes that participate in these cascades are PKA, protein kinase C (PKC), Ca2+/calmodulindependent protein kinase (CAMKII), extracellular-regulated protein kinase (ERK), phosphoinositide 3-kinase (PI3K), and glycogen synthase kinase 3 (GSK-3) [69, 70]. The downstream actions of CREB include the influences on neuron synaptic plasticity and the formation of long term memory [71, 72]. CREB is critical for the formation of hippocampally-dependent long-term memory [73]. In addition, CREB also decreases in the brains of patients with depression, which are elevated in those patients who have been using antidepressants [74].

BDNF is one of the downstream target genes of CREB, which is the most prevalent neurotrophic factor in the brain. It can be induced by the phosphorylation of CREB and responsible for neuronal survival, maintenance and growth. Some studies show that a decreased expression of BDNF, a key target implicated both in the etiology of depression [75], appears to be associated with depression symptoms both in animals and humans. Depressive patients have a decrease in serum, plasma and hippocampal BDNF levels in depressive patients [76, 77]. And a decreased serum BDNF level may be an indicator of vulnerability to develop depression [78]. On the other hand, several animal models of depression have also shown a reduced expression of BDNF in brain regions [79–82] and produce an antidepressant-like behavior [83]. Furthermore, BDNF plays important roles in facilitating both early and late phase of LTP [84, 85].

Then, CREB can induce the expression of BDNF in general [86, 87], while BDNF can also activate the production of CREB [11]. They form a positive feedback ring. BDNF is the best studied neurotrophic factor implicated in depression, which is also concerned to neuroplasticity and memory. Previous studies have found that the expression level of BDNF in the hippocampus of CUS induced mice markedly reduced [88]. BDNF also could adjust the plasticity of neurons, such as plasticity of 5-HT neurons in the central nervous system [89, 90].

It has also been indicated that the intracellular cAMP-PKA-CREB-BDNF signaling pathway can be activated after anti-depression therapy for a period. [11]. Previous studies have revealed that the cAMP-PKA-CREB -BDNF signaling is involved in depressive behaviors in animals [18, 19, 91]. Consistently, the present study showed XPJY prevented the reduction of the cAMP-PKA-CREB -BDNF signaling cascade induced by CUS while improving the depressive behaviors. The most important, the present results show the cAMP-PKA-CREB -BDNF signaling may be also involved in the ability of spatial learning memory of depressive rats. The ability of spatial cognitive recovered with the increasement of cAMP-PKA-CREB -BDNF signaling, suggesting the effects of XPJY may be through regulation of the signaling pathway. Some recent studies also supported that the ability of learning and memory improved through cAMP-PKA-CREB-BDNF signaling pathway [12, 92]. Interestingly, sertraline in the present study showed the effect of antidepression and increasement of signaling pathway as same as XPJY, but didn’t in spatial learning memory test. The XPJY 1.4 g/kg group was more advantageous than sertraline in improving the learning memory ethology of depression rats. There are two probabilities: sertraline may need more time to work, or XPJY also work though other signaling pathway about learning and memory. These studies indicated that XPJY plays antidepression and anti learning memory impairment effect through the cAMP-PKA-CREB -BDNF signaling pathway, which remains to be further studied using specific blockers of the signal pathway. Other mechanisms may also be involved, and this still requires further study.

Recently, TCM received more and more attention in treating depression and related syndromes [15–17]. XPJY is a common prescription in clinical practices and shows relatively satisfactory therapeutic effect in improving depression and cognitive dysfunction. In our previous studies, we established the depression model in the same way, and the expression of serum 5-HT and corticosterone were examined with Elisa. The results demonstrated that rats subjected to CUS exhibited decreases in serum 5-HT and increases in serum corticosterone. The results have shown that XPJY reversed the depression-like behaviors, increased serum 5-HT and decreased serum corticosterone induced by CUS in rats as the same as sertraline could [93]. In summary, the present study indicated that XPJY could improve the depression and related syndromes in rat CUS model through cAMP-PKA-CREB-BDNF pathway. These findings are important for understanding therapeutic effects of Chinese medicine XPJY on the impairment of learning and memory in depression.

## Conclusions

XPJY has regulatory effect on depressive behavior in the sucrose preference and open field exploration, and learning and memory in Morris water maze, and the expression of cAMP, PKA, CREB and BDNF in hippocampus of model rats. The effect is probably achieved mainly by activating cAMP-PKA-CREB-BDNF signaling pathway. This study provides experimental evidence for the clinical application of XPJY in the treatment of depression and related learning and memory impairment.

## Abbreviations

BDNF: Brain derived neurotrophic factor
cAMP: Cyclic adenosine monophosphate
CREB: CAMP response element-binding protein
CUS: Chronic unpredictable stress
MWM: Morris water maze
PKA: Protein kinase A
rDD: Recurrent depressive disorder
SSRI: Selective serotonin reuptake inhibitors
TCM: Traditional Chinese medicine
XPJY: XingPiJieYu decoction
